# Dehydrated Human Amnion–Chorion Membrane as a Bioactive Scaffold for Dental Pulp Tissue Regeneration

**DOI:** 10.3390/biomimetics9120771

**Published:** 2024-12-18

**Authors:** Sahng G. Kim

**Affiliations:** Division of Endodontics, Columbia University College of Dental Medicine, New York, NY 10032, USA; sgk2114@cumc.columbia.edu

**Keywords:** amnion–chorion membranes, dental pulp regeneration, regenerative endodontics, bioactive scaffolds, biomaterials

## Abstract

The dehydrated human amnion–chorion membranes (dHACMs) derived from the human placenta have emerged as a promising biomaterial for dental pulp regeneration owing to their unique biological and structural properties. The purpose of this review is to explore the potentials of dHACMs in dental pulp tissue engineering, focusing on their ability to promote cellular proliferation, differentiation, angiogenesis, and neurogenesis. dHACMs are rich in extracellular matrix proteins and growth factors such as TGF-β1, FGF2, and VEGF. They also exhibit significant anti-inflammatory and antimicrobial properties, creating an optimal environment for dental pulp regeneration. The applications of dHACMs in regenerative endodontic procedures are discussed, highlighting their ability to support the formation of dentin and well-vascularized pulp-like tissue. This review demonstrates that dHACMs hold significant potential for enhancing the success of pulp regeneration and offer a biologically based approach to preserve tooth vitality and improve tooth survival. Future research is expected to focus on conducting long-term clinical studies to establish their efficacy and safety.

## 1. Introduction

Dental pulp regeneration is a rapidly advancing area of research in regenerative dentistry, focused on restoring the natural function and vitality of dental pulp that has been compromised by trauma and infection [1,2,3]. Unlike traditional endodontic treatments, which aim to remove the infected pulp tissue and replace it with bioinert materials, dental pulp regeneration seeks to restore the natural tissue, preserving the tooth’s vitality [4,5,6,7]. The preservation of vitality is a key benefit of regenerative treatments, which work by encouraging the body’s natural healing processes [3,4,6]. The challenges in dental pulp regeneration primarily revolve around creating an optimal environment for survival and differentiation. Specifically, there is a need to foster the proliferation of stem/progenitor cells, stimulate angiogenesis, and ensure proper innervation to restore normal tooth function [5]. Traditional dental materials used in endodontics, such as gutta-percha and sealers, are not conducive to these regenerative processes [5]. Therefore, there is a growing interest in developing biomaterials that not only maintain the structural integrity of a tooth, but also actively promote tissue regeneration.

One promising biomaterial in this regard is the human amnion–chorion membrane (ACM), derived from the innermost layers of the human placenta [8,9]. ACM has been used in various medical fields owing to its unique composition of bioactive molecules, including growth factors and collagen, which aid tissue healing and regeneration [10,11,12]. ACM consists of two layers: the amnion and the chorion, both of which are known for their rich bioactive molecule content [13,14,15]. Through dehydration after the cleansing and lamination of the amnion and chorion under controlled conditions, ACM can be transformed into a dehydrated human amnion–chorion membrane (dHACM) [10,11,12]. dHACMs have an extended shelf life and are easier to store and handle while preserving their biocompatibility and therapeutic properties [11]. These properties make dHACMs attractive candidates for use in dental pulp regeneration.

The use of dHACMs in regenerative endodontics presents a number of advantages. Such dHACMs provide a scaffold that supports cell attachment, proliferation, and differentiation, while also possessing anti-inflammatory properties that can modulate the immune response and promote healing [10,11,12]. Given these properties, dHACMs may overcome many of the barriers to effective dental pulp regeneration, particularly in relation to promoting the complex biological processes needed for full tissue regeneration.

Several review articles have explored the use of dHACMs in regenerative medicine for treating traumatic injuries or chronic skin wounds, suggesting that dHACMs may improve wound healing and decrease the healing time compared to traditional treatments [10,16,17,18,19]. There is also a recent review article discussing various forms of amniotic membranes used in endodontic care, but it includes little discussion around dHACMs [20]. Despite the recognized potential of dHACMs in this field, no comprehensive review has yet investigated their use for dental pulp tissue regeneration. Therefore, the aim of this narrative review is to explore and assess the potential applications of amnion–chorion membranes, specifically dehydrated human amnion–chorion membranes (dHACMs), in dental pulp tissue regeneration. Given the nascent stage of dHACMs in regenerative endodontics, there are limited studies specifically targeting dental pulp regeneration. This review synthesizes the existing research on the biological properties of dHACMs, their mechanisms for promoting tissue regeneration, and their developing clinical applications in regenerative endodontics. Additionally, it emphasizes the vital role that biomaterials like dHACMs could play in overcoming the current challenges in dental pulp regeneration, offering a more biologically harmonious approach compared to traditional methods.

## 2. Literature Search

Electronic searches were performed in the PubMed, Scopus, Web of Science, Embase, and Medline databases from their inception until July 2024 to identify studies examining the use of dHACMs for dental pulp tissue regeneration. Relevant articles were identified using combinations of the following keywords: ‘amnion–chorion membrane’, ‘dehydrated human amnion–chorion membrane’, ‘pulp regeneration’, ‘pulp tissue engineering’, ‘pulp engineering’, ‘pulp revascularization’, ‘pulp revitalization’, and ‘regenerative endodontics’. The inclusion criteria consisted of in vitro studies, animal studies, prospective studies, retrospective studies, case series, and case reports. Additionally, animal studies examining the properties of dHACMs were included to gain insights into the mechanism of action in relation to tissue regeneration. Other relevant articles exploring the biological properties and mechanisms of dHACMs in the context of tissue engineering were also included to enrich the scope of this comprehensive review.

This review is intended as a comprehensive review rather than a systematic review, aiming to provide a detailed synthesis of the existing literature on the subject.

## 3. Biological Composition and Properties

### 3.1. Structural Components of Amnion–Chorion Membranes

**Figure 1:**
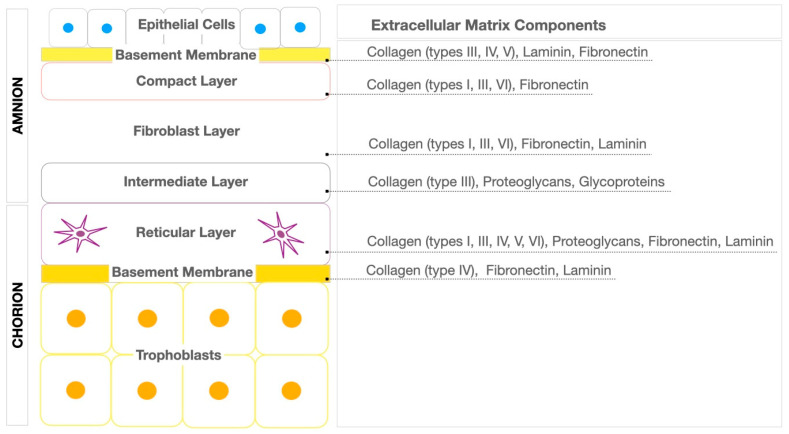
Schematic diagram illustrating the structure of the amnion–chorion membrane and its detailed extracellular matrix components. The diagram highlights the distinct layers of the membrane—amnion and chorion—showing their unique composition and the types of collagen fibers, proteoglycans, glycoproteins, fibronectin, and laminin.

The amnion–chorion membrane is a biologically derived structure composed of two distinct layers that work together to provide structural integrity and biological function (Figure 1). The innermost layer, known as the amnion, is a thin, avascular membrane characterized by a high concentration of collagen fibers, including types I, III, IV, V, and VI, alongside other important components such as laminin, fibronectin, and glycosaminoglycans [12,14,21,22,23]. These molecules form a highly organized and resilient extracellular matrix (ECM) which plays a crucial role in supporting cellular adhesion, proliferation, and migration [12,14,21,22,23]. The matrix is essential for the tissue’s ability to facilitate the regeneration of various cell types and tissues. The outer layer, the chorion, is thicker and more fibrous compared to the amnion, offering enhanced structural support. The chorion contains similar ECM molecules, such as collagen types I, III, IV, V, and VI, proteoglycans, laminin, and fibronectin, which contribute to its functionality [12,14,21,22,23,24]. The chorion’s fibrous nature not only strengthens the overall structure of the membrane, but also complements the properties of the amnion, ensuring that the membrane remains strong yet flexible.

The dual-layer configuration of the amnion and chorion provides a membrane that is uniquely suited for a variety of regenerative applications [12,14,15]. Its combination of strength, flexibility, and biologically active components allows it to facilitate healing and regeneration in numerous tissues. The structural integrity of the amnion–chorion membrane, along with its supportive ECM, makes it promising for use in dental pulp regeneration.

### 3.2. Processing and Low Immunogenicity of dHACMs

The low immunogenicity of dHACMs is largely attributed to their specialized processing methods, which are designed to preserve the biological properties of the tissue while minimizing its antigenic potential [16,25,26]. One of the key processing techniques involves the gentle cleansing of the amnion and chorion layers to remove contaminating materials while preserving the essential biological factors [25]. This cleaning procedure is designed to eliminate cellular debris, bloodborne pathogens, and potential immunogenic proteins without causing significant damage to the tissue’s structural integrity. The gentle nature of this cleansing procedure ensures that the inherent growth factors, which play critical roles in wound healing and tissue regeneration, are retained. The dehydration of the amnion and chorion layers is another crucial step in the processing of dHACMs [26]. Controlled dehydration is conducted under stringent conditions that ensure the longevity and stability of the graft. This process not only helps preserve the matrix structure, but also maintains the functionality of the remaining biological molecules. By removing the water content, the likelihood of microbial growth and immune recognition is significantly reduced, further contributing to the low immunogenicity of dHACMs. The processing methods of dHACMs generally involve the removal of viable cells and their associated immunogenic components [16,25,26]. Most processing protocols aim to minimize cellular and nuclear antigens, which are often the primary targets for immune responses [26,27]. By removing these cellular elements, the risk of triggering an immune response upon implantation is substantially reduced. The resulting product consists, predominantly, of an acellular matrix rich in extracellular matrix components and bioactive molecules, helping reduce the potential for immune rejection. Although the cellular components are largely removed, processing methods retain key biological factors, such as growth factors and cytokines. These molecules are pivotal to promote healing and tissue integration, and their preservation is achieved through careful processing that avoids chemical alteration. The retained factors can contribute to the promotion of a favorable healing environment without eliciting significant immune reactivity, thereby enhancing the therapeutic efficacy of dHACMs.

### 3.3. Bioactive Molecules: Growth Factors, Cytokines, and ECM Proteins

dHACMs are a rich source of bioactive molecules that are integral to the process of tissue regeneration. The membrane is embedded with a wide array of growth factors, which play essential roles in promoting cellular proliferation, differentiation, and tissue repair. Among these are the transforming growth factor (TGF-β), the fibroblast growth factor (FGF), the platelet-derived growth factor (PDGF), the epidermal growth factor (EGF), the vascular endothelial growth factor (VEGF), the placental growth factor (PIGF), the hepatocyte growth factor (HGF), and the granulocyte colony-stimulating factor (GCSF) [25,28,29,30]. Each of these growth factors contributes uniquely to the regenerative process, helping stimulate the growth of cells, blood vessels, and tissues critical for successful healing and restoration.

In addition to these growth factors, dHACMs are also enriched with various cytokines such as interleukins (IL) and tissue inhibitors of metalloproteinases (TIMP). These molecules help modulate the immune response, thereby reducing inflammation and creating a more favorable environment for tissue regeneration [29]. By tempering the inflammatory process, cytokines help prevent excessive tissue damage while simultaneously promoting healing. This anti-inflammatory action is particularly important in regenerative applications, where the minimization of chronic inflammation can lead to better outcomes.

ECM proteins found in dHACMs, such as collagen, laminin, fibronectin, and glycosaminoglycans [25,28,29], may further enhance the membrane’s regenerative potential. These ECM components provide structural support to the tissue and serve as a scaffold for cell attachment and migration. Additionally, they create an environment that is conducive to cellular activities that are crucial for tissue regeneration, such as cell proliferation and differentiation. These ECM proteins not only reinforce the physical properties of the membrane, but also contribute to biochemical signaling, which is vital for tissue development and repair.

The regenerative effects of the bioactive molecules contained in dHACMs are summarized in Table 1, where their roles in promoting dental pulp regeneration and tissue healing are described.

### 3.4. Antimicrobial and Immunomodulatory Properties

dHACMs exhibit significant antimicrobial properties, which are important in a microenvironment where infection control is critical. In the context of dental pulp regeneration, controlling infections is crucial to ensure successful outcomes, and dHACMs play a role in this by inhibiting the growth of common oral pathogens. Specifically, dHACMs have been shown to suppress the proliferation of bacteria such as *Streptococcus mutans* and *Streptococcus oralis*, two species commonly associated with oral infections. This antimicrobial activity helps reduce the risk of infection both during and after pulp regeneration procedures, creating a safer environment for tissue healing and regeneration [29].

The antimicrobial effects of dHACMs have been demonstrated through various in vitro studies confirming its ability to combat multiple bacterial strains [35,36,37,38]. These studies suggest that the membrane secretes antimicrobial peptides such as beta-defensins and elafin, both of which are known for their capacity to neutralize bacterial threats [18,19]. These peptides actively work to prevent bacterial colonization, thus enhancing the membrane’s utility in dental pulp regeneration where infection control is critical.

In addition to their antimicrobial properties, dHACMs also exhibit robust immunomodulatory functions. This is achieved through the secretion of various cytokines and regulatory molecules, including the IL-1 receptor antagonist, IL-4, IL-6, IL-8, IL-10, TIMP-1, TIMP-2, and TIMP-4 [25,29,32,33,34]. These molecules help regulate the immune response by reducing inflammation and promoting a balanced healing environment. By modulating the immune system, dHACMs not only foster tissue regeneration, but also minimize the risk of chronic inflammation that could impair the healing process.

Furthermore, the membrane’s low immunogenicity makes it highly compatible for clinical use [11,23]. Being derived from human tissue and possessing immunoprivileged status, dHACMs are less likely to provoke an adverse immune response, such as graft rejection. This natural compatibility allows the membrane to integrate more effectively with the host tissue, leading to improved healing and regeneration outcomes without the complications that may arise from immune rejection [11,39].

### 3.5. Biomechanical Properties Relevant to Pulp Regeneration

The biomechanical properties of dHACMs are critical to their successful application in dental pulp regeneration. Their inherent tensile strength and flexibility enable the membrane to conform closely to the complex anatomy of the root canal system [11]. This adaptability is crucial, as it allows dHACMs to provide a stable and supportive scaffold that facilitates the growth and regeneration of pulp tissue within the canal. The composition and biomechanical properties of dHACMs compared to natural dental pulp are summarized in Table 2.

Moreover, the versatility of dHACMs is enhanced when micronized, a process that reduces the membrane to small particles, allowing it to be more easily delivered to the root canal system. When combined with a hydrogel, micronized dHACMs exhibit even better adaptability to the root canal’s irregular spaces and shapes, ensuring a more uniform coverage and a closer contact with the canal walls [50]. This improves the overall regenerative potential by creating an optimal microenvironment for tissue growth and regeneration.

Another important feature of dHACMs is their resorbability. As the membrane gradually degrades over time, it is naturally replaced by newly formed tissue. This controlled resorption ensures that the membrane does not interfere with the natural healing process but, instead, complements it. The gradual breakdown of the membrane provides a continuous source of bioactive molecules that promote regeneration while avoiding the need for removal or causing any adverse effects associated with long-term biomaterial presence [51].

## 4. Mechanisms of Action in Dental Pulp Regeneration

### 4.1. Interaction of dHACMs and Stem Cells

The interaction between dHACMs and stem cells is fundamental to a successful pulp regeneration. When dHACMs are used in pulp regeneration, they serve as a biologically active scaffold that supports the attachment, proliferation, and differentiation of the stem cells, either transplanted exogenously or homed from the periapical tissues. The presence of dHACMs creates a supportive framework that encourages stem cells to migrate to the site of injury or tissue defect within the root canal system, establishing a foundation for new tissue growth.

The ECM components of dHACMs, such as collagen, fibronectin, and laminin [16,24,25,28,29,52], play a key role in this regenerative process. These ECM proteins closely resemble the natural ECM found in the dental pulp, creating a conducive microenvironment that enhances cellular activities [25,28,29]. The similarity to native dental pulp ECM not only promotes the adhesion of stem cells to the membrane, but also provides guidance cues that facilitate cell migration to the desired location [25,28,29,46]. This ECM-mimicking environment is vital to create the optimal conditions for stem cells to thrive and initiate the repair process.

Moreover, dHACMs are rich in growth factors, which are instrumental for guiding stem cell differentiation into odontoblast-like cells [11,25,28,29,53,54]. These cells are specialized for dentinogenesis in the root canal system. Growth factors such as TGF-β1, FGF2, and PDGF-bb signal to the stem cells, directing them to adopt an odontoblastic phenotype. This differentiation process is essential for the restoration of the tooth’s structural integrity, as the newly formed odontoblast-like cells contribute to the regeneration of the pulp–dentin complex.

### 4.2. Role of dHACMs in Angiogenesis

Angiogenesis is a critical process during tissue regeneration, as it ensures that newly formed tissues receive adequate nutrients and oxygen. In the context of dental pulp regeneration, angiogenesis is particularly crucial to support the vitality and functionality of the regenerated tissue. dHACMs facilitate this process by promoting the proliferation of endothelial cells, which are essential for forming new blood vessels within the root canal system [28,55,56,57]. This revascularization process plays a key role in establishing a robust blood supply to the regenerating pulp tissue.

The angiogenic potential of dHACMs is associated with their rich composition entailing growth factors and signaling molecules that drive vascular formation. Key angiogenic factors present in dHACMs include VEGF, PDGF-bb, PIGF, HGF, angiotensin, and angiopoietin-2 [14,25,28]. These molecules stimulate endothelial cell migration and proliferation, thereby encouraging the formation and maturation of blood vessels in the treated area. As new blood vessels develop within the root canal, the regenerated pulp tissue gains access to a continuous supply of nutrients and oxygen, both of which are essential for sustaining cellular activities and preventing necrosis [4].

The revascularization not only supports the structural regeneration of the dental pulp, but also contributes to the functional restoration of the tissue [6,54]. By establishing a healthy blood supply, dHACMs ensure that the regenerated pulp remains vital and capable of responding to physiological stimuli, a key criterion for successful regenerative endodontic treatments. The ability to promote both structural and functional regeneration makes dHACMs promising candidates for regenerative endodontics, where the ultimate goal is to restore the natural function and longevity of a tooth.

### 4.3. ECM Remodeling and Scaffold Function of dHACMs

dHACMs play a pivotal role in the ECM remodeling process during dental pulp regeneration. The ECM is critical for providing structural support to cells and for regulating various cellular functions, including adhesion, migration, and differentiation. As a scaffold, dHACMs mimic the natural ECM of the dental pulp, creating an environment that promotes these cellular processes and encourages the growth of new tissue within the root canal system [12,14,25].

Once the dHACM scaffold is inserted into the root canal space, it becomes a dynamic structure that interacts with the host cells. The scaffold is gradually remodeled by the host cells. Matrix metalloproteinases produced by the host cells break down the components of dHACMs. This controlled breakdown facilitates the infiltration of host endogenous cells from the surrounding tissues and encourages the deposition of host-derived ECM proteins within the scaffold [58]. As the dHACMs gradually degrade, they are replaced by a newly formed ECM that is integrated with the host tissue, allowing the regenerated tissue to become part of the native dental structure.

This ECM remodeling process, facilitated by the dHACMs, ensures that the scaffold is not only a temporary support structure, but also an integral part of the healing process [56,57]. The controlled degradation of dHACMs can be tailored by adjusting the properties of the membranes to meet specific clinical needs [58]. By controlling its degradation rate, the scaffold can provide support during the critical early stages of tissue regeneration, helping stabilize the forming tissue [57,58]. As regeneration progresses, the scaffold gradually breaks down, allowing for complete integration of the regenerated pulp tissue with the existing dental structures, ultimately restoring the tooth’s structural integrity and function.

### 4.4. Anti-Inflammatory Function and Wound Healing Promotion

The anti-inflammatory properties of dHACMs are essential to regulate the inflammatory response during dental pulp regeneration. While inflammation is a natural and necessary part of the healing process [59], excessive or prolonged inflammation can hinder regeneration and lead to pulp necrosis, ultimately causing the failure of regenerative treatments [4]. Therefore, controlling the inflammatory response is crucial for a successful dental pulp regeneration.

dHACMs contribute to inflammation management through the release of specific cytokines and regulatory proteins, such as IL-1 receptor antagonist, IL-4, IL-10, and TIMP. The IL-1 receptor antagonist inhibits the proinflammatory effects of IL-1 by binding to IL-1 receptors without activating them. This competitive binding prevents IL-1 from interacting with its receptors, effectively blocking its ability to initiate inflammatory responses [25]. IL-4 and IL-10 are known for their anti-inflammatory properties, as they work to suppress the production of proinflammatory mediators and reduce the recruitment of immune cells to and their infiltration into the injury site [29]. TIMP proteins also play a role in modulating inflammation by inhibiting matrix metalloproteinases, which can otherwise exacerbate tissue breakdown and inflammation if left uncontrolled [29,59,60,61]. Through these actions, dHACMs help create a balanced inflammatory response, allowing for an environment that is more conducive to tissue healing and regeneration.

By controlling the level and duration of inflammation, dHACMs reduce the risk of complications associated with excessive immune activity, such as tissue damage and delayed healing [60,61,62]. This balanced inflammatory environment may not only support a faster recovery, but also improve the likelihood of successful tissue regeneration by allowing stem cells and other regenerative factors to function optimally within the root canal space.

Additionally, the presence of antimicrobial properties in ACMs [29,32,33,34,39,63,64,65] further supports the healing process by reducing the risk of infection, which is a common challenge in endodontic procedures. By suppressing microbial growth, dHACMs help prevent infections that could otherwise trigger an excessive immune response, disrupt the regeneration process, or lead to treatment failure. The anti-inflammatory and antimicrobial properties of dHACMs work synergistically to create a favorable environment for dental pulp regeneration. By controlling inflammation and reducing the risk of infection, dHACMs can promote faster and more efficient healing.

## 5. In Vitro and In Vivo Studies Using dHACMs

### 5.1. Cellular Responses to dHACMs

In vitro and in vivo studies have provided valuable insights into the cellular responses to ACMs and their potential for use in dental pulp regeneration. An in vitro study by Bang et al. [53] demonstrated that dHACMs can significantly enhance the migration of human dental pulp stem cells and human umbilical vein endothelial cells. This migration is essential for tissue regeneration, as it facilitates the movement of key cells to the site of injury or tissue defect. Additionally, the membranes exhibited a high potential for mineralization, as evidenced by their high alkaline phosphatase (ALP) activity, the formation of mineral nodule, and the upregulated expression of osteogenic and dentinogenic genes, including ALP, OSX, and DMP1 [53]. These markers suggest that dHACMs can promote mineralized tissue formation, which is critical for regenerating the pulp–dentin complex.

Moreover, dHACMs has been shown to upregulate angiogenic genes and increase capillary tube formation, which is indicative of enhanced angiogenesis [53]. Angiogenesis is essential for supplying nutrients and oxygen to the regenerating pulp tissue. The study also found that dHACMs decreased the expression of proinflammatory genes such as TNF-α, IL-1β, and IL-6 [53], thereby helping create an anti-inflammatory environment conducive to healing.

The antimicrobial properties of dHACMs have also been demonstrated in several studies [29,35,36,37,38,63,64,65]. In one in vitro study by Palanker et al. [64], dHACMs were found to completely eliminate *Streptococci gordonii*, a bacterial species commonly found in oral infections. Another in vitro study by Ashraf et al. [65] demonstrated that the antibacterial effects of dHACMs against oral bacterial species are comparable to those achieved with a tetracycline treatment, suggesting that dHACMs may offer effective infection control without the need for antibiotics.

In vivo studies further support the regenerative potential of dHACMs [28,29]. By using subcutaneous implantation models, it was observed that significantly more hematopoietic stem cells [28] and mesenchymal stem cells [29] were recruited to the membrane implant site compared to a control skin site. The recruitment of these stem cells is crucial for tissue regeneration, as they provide a source of cells capable of differentiating into various cell types necessary for tissue repair.

### 5.2. Efficacy of dHACMs in Pulp Regeneration

Animal studies serve as a critical bridge for the translation of in vitro findings to potential clinical applications, providing insights into how materials and techniques perform in a living system. In a study by Kim and Solomon [66] using a dog model, three different regenerative procedures—blood clot formation, collagen membranes, and dHACMs—were evaluated in terms of their effectiveness in promoting pulp regeneration. This comparative study aimed to assess which material could best support cell homing-based pulp regeneration in mature teeth (Table 3).

Histological analyses of the treated teeth revealed significant differences among the groups [66]. The dHACM group demonstrated more extensive intracanal fibrous tissue formation, a greater presence of odontoblast-like cells, and a lower degree of periapical inflammation compared to the groups treated with blood clots or collagen membranes. The increased presence of odontoblast-like cells in the dHACM group indicates enhanced differentiation and organization of cells essential for dentin formation. Furthermore, the reduced periapical inflammation observed in the dHACM group suggests a more favorable immune response, which is critical for maintaining a stable environment conducive to regeneration.

**Figure 2:**
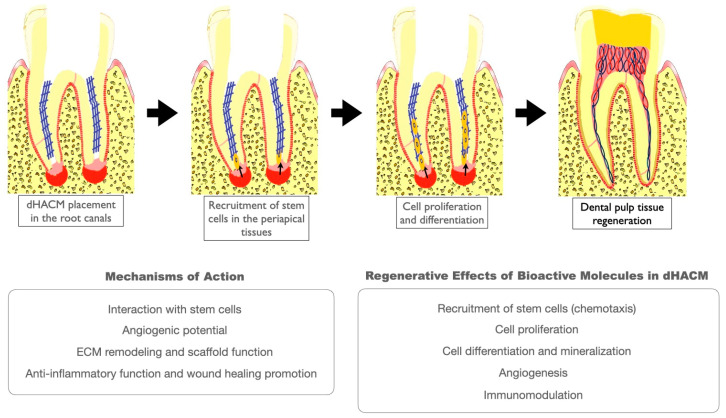
Cell homing-based pulp regeneration using dHACMs. dHACMs promote the recruitment of stem cells from the periapical tissue (e.g., stem cells of the apical papilla, bone marrow stem cells, periodontal ligament stem cells) and augment cellular events which are critical for pulp regeneration, such as angiogenesis, odontoblast differentiation, cell proliferation, and immunomodulation. Moreover, the membranes possess anti-inflammatory and antimicrobial properties and enhance wound healing and ECM remodeling through their function as a bioactive scaffold.

These findings underscore the potential of dHACMs as effective scaffolds for cell homing-based pulp regeneration. By supporting fibrous tissue formation, promoting the differentiation of odontoblast-like cells, and minimizing inflammatory responses, dHACMs show promise as regenerative materials for mature teeth, where traditional pulp regeneration techniques may be less effective. The results from this animal study highlight dHACMs’ advantages over conventional regenerative materials, suggesting that they could offer a more viable option for clinical applications in endodontic treatments aimed at restoring pulp vitality and function using a cell homing approach (Figure 2).

## 6. Applications in Dental Pulp Regeneration

### 6.1. The Use of dHACMs in Regenerative Endodontic Procedures (REPs)

**Figure 3:**
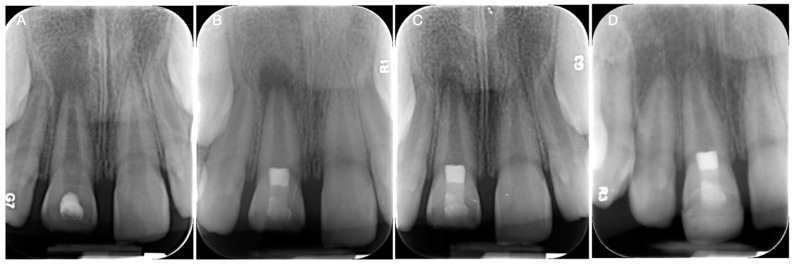
Clinical case using dHACMs for pulp regeneration. (**A**) Preoperative radiograph showing the maxillary right central incisor with a periapical lesion. The tooth was diagnosed with previously initiated therapy and symptomatic apical periodontitis (**B**) Postoperative radiograph after root canal disinfection, placement of dHACMs, and restoration. (**C**) Three-month follow-up showing a reduction in size of the periapical lesion. (**D**) Fourteen-month follow-up showing the complete resolution of the periapical lesion and the complete root formation.

Regenerative endodontic procedures (REPs) are advanced treatments aimed at regenerating the pulp–dentin complex in teeth with necrotic pulp and immature roots [4,67,68]. REPs involve the disinfection of the root canal system, followed by the placement of a scaffold and the induction of bleeding to introduce stem cells into the canal [4,68]. The goal is to stimulate the regeneration of the pulp tissue, allowing the tooth to continue root development and maintain its vitality [67,68]. dHACMs can serve as effective scaffolds in REPs, providing a structure that supports the attachment, proliferation, and differentiation of stem cells within the root canal [14,25,28,53]. The membranes’ bioactive properties enhance the regenerative process by promoting angiogenesis [14,25,28,55,56,57] and dentinogenesis [66]. Additionally, ACMs’ anti-inflammatory [25,29,32,33,34] and antimicrobial effects [29,35,36,37,38,63,64] help create a favorable environment for tissue regeneration, reducing the risk of infection and inflammation. A clinical case using dHACMs as scaffolds for pulp regeneration is presented in Figure 3.

### 6.2. The Use of dHACMs in Vital Pulp Therapy

**Figure 4:**
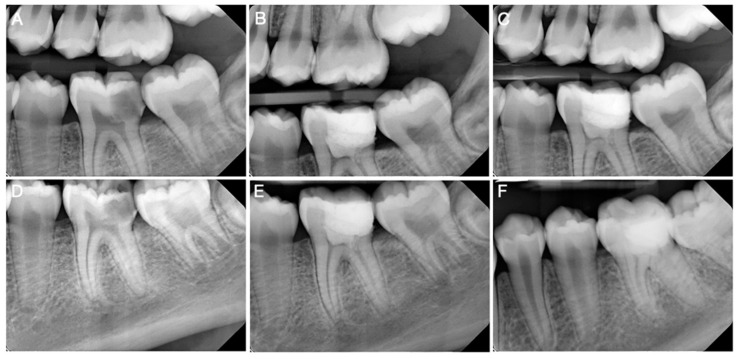
Clinical case using dHACMs for vital pulp therapy. (**A**,**D**) Preoperative radiograph showing the mandibular left first molar with extensive caries involving the distal pulp horn. The tooth was diagnosed with symptomatic irreversible pulpitis and symptomatic apical periodontitis. (**B**,**E**) Postoperative radiograph after the removal of both caries and inflamed coronal pulp tissue, the placement of dHACMs, and restoration. (**C**,**F**) Three-month follow-up showing no periapical lesion. The tooth was negative to percussion and palpation and positive to electric pulp testing.

Vital pulp therapy (VPT) is a treatment approach aimed at preserving the vitality of the dental pulp after it has been exposed or nearly exposed due to caries, trauma, or restorative procedures [69,70,71,72]. The goal of VPT is to maintain the health of the remaining pulp tissue and stimulate the formation of new dentin, thereby protecting the pulp from further damage [73,74]. dHACMs can be used in VPT as biologically active dressings that cover the exposed pulp and promote healing and regeneration. The membranes’ content in terms of growth factors [25,28,29,30] and ECM proteins [25,28,29] supports the formation of reparative dentin, which seals off the pulp and prevents bacterial infiltration. Additionally, dHACMs’ anti-inflammatory properties may help control the inflammatory response, reducing the risk of pulpitis and necrosis [59,60,61,62]. A clinical case using dHACMs for vital pulp therapy is presented in Figure 4.

## 7. Challenges and Limitations

### 7.1. Variability in Biological Composition

One of the challenges associated with the use of dHACMs is the potential variability in their biological composition. As a natural material derived from human tissue, the composition of dHACMs can vary depending on the donor’s age, health, and genetic factors. This variability can affect the concentration of bioactive molecules, such as growth factors and cytokines, which are critical for tissue regeneration. Additionally, ensuring consistency across different batches of dHACMs can be challenging. This variability may affect the consistency of clinical outcomes, making it important to standardize the processing and handling of the membrane to ensure reliable results.

### 7.2. Potential for Immune Response

dHACMs are widely recognized for their low immunogenicity, a characteristic attributed to their specialized processing methods [16,25,26] and their inherent immunoprivileged status [75,76]. This means that dHACMs are less likely to provoke a significant immune reaction when introduced into the body. The harvesting and preparation processes for dHACMs are designed to minimize antigenicity, thereby reducing the likelihood of rejection by the recipient’s immune system. As a result, they have been effectively used in various clinical settings, particularly for the treatment of chronic wounds and soft-tissue injuries, where their use promotes healing and tissue regeneration with minimal inflammatory responses [76,77,78,79,80]. However, despite the consensus on their low immunogenic profile, it is essential to acknowledge that there exists a minor but notable risk of immune response in specific patient populations. Individuals with highly sensitive immune systems, such as those with autoimmune disorders or individuals undergoing immunosuppressive treatments, may exhibit atypical reactivity to dHACMs [81]. In such cases, even a product regarded as immunologically safe could potentially elicit a localized or systemic immune response. This response could manifest as inflammation or other complications that may compromise the efficacy of the treatment and the healing process.

### 7.3. Handling and Application Challenges

The handling and placement of a dHACM within the root canal space can be challenging. Its flexibility requires clinicians to handle it carefully and positioning it precisely within the complex anatomy of the root canal system can be difficult. Cutting the membrane into smaller pieces or processing it into a micronized powder form improve its adaptability to the root canal; however, preparing these modified forms can be intricate and time-consuming.

Since the use of dHACMs is relatively new in pulp regeneration, there are currently no standardized guidelines on dosage, preparation, and application, resulting in inconsistent practices among clinicians. Additionally, the learning curve for practitioners unfamiliar with the handling of dHACMs may limit their widespread adoption and could impact treatment outcomes.

### 7.4. Limited Long-Term Clinical Evidence

While in vitro and short-term in vivo studies have shown promising results for dHACM use in regenerative applications, there is limited long-term data on its efficacy in dental pulp regeneration. More extensive studies are needed to understand how dHACMs perform in clinical settings over time, including their impact on tissue stability, functional outcomes, and overall success in pulp regeneration.

## 8. Conclusions

dHACMs present significant potential as a biomaterial for dental pulp regeneration, offering a biologically active scaffold that supports key cellular processes such as adhesion, migration, proliferation, and differentiation. Their bioactive properties—including the presence of growth factors, anti-inflammatory cytokines, and antimicrobial peptides—make them uniquely suited for regenerative endodontics. They provide a conducive microenvironment for tissue regeneration, reducing inflammation, and minimizing infection risks.

This review highlights the unique biological composition and bioactivity of dHACMs which allow them to facilitate essential regenerative processes such as angiogenesis, extracellular remodeling, and immunomodulation. These properties not only support the formation of new pulp tissue, but also contribute to the restoration of pulp vitality and function, distinguishing dHACMs from traditional endodontic materials that lack regenerative potential.

However, several challenges remain, including variability in biological composition, handling difficulties, and a lack of standardized protocols. Furthermore, long-term clinical data on dHACMs’ efficacy in dental pulp regeneration are limited, underscoring the need for further research to evaluate their durability, integration with host tissues, and overall impact on treatment outcomes. Addressing these challenges is crucial to fully realize dHACMs’ potential as a transformative biomaterial in regenerative endodontics, paving the way for treatments that not only restore structural integrity but also revise the natural function of dental pulp.

## Figures and Tables

**Table 1:** Regenerative effects of the bioactive molecules in dHACMs.

Regenerative Effects	Bioactive Molecules
Recruitment of stem cells (chemotaxis) [15,25,28]	TGF-β1, FGF-2, GCSF, HGF
Cell proliferation [14,25,28]	TGF-β1, FGF2, GCSF, EGF, PDGF, HGF
Cell differentiation and mineralization [31]	TGF-β1, FGF2, PDGF-bb
Angiogenesis [14,25,28]	VEGF, PDGF-bb, PIGF, HGF, angiotensin, angiopoietin-2
Immunomodulation [25,29,32,33,34]	IL-1 receptor antagonist, IL-4, IL-6, IL-8, IL-10, TIMP-1, TIMP-2, TIMP-4

**Table 2:** Composition and biomechanical properties of dHACMs compared to natural dental Ppulp.

	dHACM	Natural Dental Pulp
**Composition**		
Primary extracellular components	Collagen (types I, III, IV, V), fibronectin, and laminin [25,28,29].	Collagen (types I, III, V, VI) [31,40,41,42,43,44,45,46].
Cellular components	Nonviable cells [25].	Fibroblasts, odontoblasts, immune cells, and stem cells [31,45,46].
Other components	Growth factors, cytokines, and tissue inhibitors of metalloproteinases, proteoglycan, and glycosaminoglycan [25,28,29].	Proteoglycan, glycosaminoglycan, non-collagenous proteins (fibronectin, osteonectin), blood vessels, and nerves [31,46].
**Biomechanical properties**		
Tensile strength	Higher than natural dental pulp, but varies with hydration and decellularization methods [25].	Low and high water content and cellular components [47].
Compressive strength	Low and soft connective tissue.	Low and soft connective tissue.
Viscoelasticity	Viscoelastic [48].	Viscoelastic [49].

**Table 3:** Summary of published studies using dHACMs for dental pulp regeneration.

Study	Study Types	Methods	Main Findings	Clinical Implications
Bang et al., 2022 [53]	In vitro	ProRoot MTA, RetroMTA, collagen membrane, and dHACMs were included. - Transwell and scratch assays were used for cell migration and wound healing. - Mineralization potential was assessed through alkaline phosphatase activity, Alizarin red S staining, and qRT-PCR. Angiogenesis was evaluated by using an endothelial tube formation assay.	Membranes including collagen membranes and dHACMs demonstrated greater migration and wound healing compared to ProRootMTA and RetroMTA. A high mineralization potential and alkaline phosphatase activity were observed with ProRootMTA and both membranes. Both membranes facilitated an increased gene expression for angiogenesis and promoted capillary tube formation.	dHACMs and collagen membranes effectively support cell migration and angiogenesis, and have a mineralization potential comparable to that of calcium–silicate materials, highlighting their suitability as scaffolds for pulp regeneration.
Kim and Solomon, 2021 [66]	In vivo	Twenty-four roots from mature canine premolars were equally divided into three groups: blood clots (BC), collagen membranes (CM), and dHACMs. - Root canal disinfection with 1.5% NaOCl and calcium hydroxide was performed following root canal infection. - After two weeks, bleeding was promoted to create blood clots or membrane insertion. - Histological assessment after 12 weeks.	Fibrous connective tissue was present in all groups, most predominatly in the dHACM group. Odontoblast-like cells were found only in the dHACM group. Intracanal mineralized tissue was observed only in the BC and CM groups. More periapical inflammation was observed in the BC group than in the dHACM group.	dHACMs may be useful for cell homing-based pulp regeneration in mature teeth.

## Data Availability

Not applicable.
